# Biological aging mediates and frailty modifies the associations of arterial stiffness with incident probable sarcopenia and sarcopenia: a prospective cohort study of UK biobank

**DOI:** 10.3389/fpubh.2026.1926399

**Published:** 2026-09-14

**Authors:** Aochuan Sun, Yike Du, Xusheng Yang, Hao Liu, Ruowei Zeng, Yang Wu, Zhengtang Liu

**Affiliations:** 1 Department of Geriatrics, Xiyuan Hospital, China Academy of Chinese Medical Sciences, Beijing, China; 2 Graduate School, Beijing University of Chinese Medicine, Beijing, China; 3 Department of Nephrology, Beijing Rehabilitation Hospital, Capital Medical University, Beijing, China

**Keywords:** arterial stiffness, biological age acceleration, frailty, sarcopenia, UK biobank

## Abstract

**Background:**

Arterial stiffness may contribute to sarcopenia, but whether biological aging mediates and frailty modifies these associations remains unclear.

**Methods:**

This prospective cohort study included 32,451 UK Biobank participants free of probable sarcopenia and sarcopenia at baseline. Cause-specific Cox proportional hazards models were used to examine the associations between arterial stiffness index (ASI) and incident probable sarcopenia and sarcopenia. Restricted cubic spline analyses were performed to characterize dose–response relationships. Counterfactual mediation analysis was used to evaluate the mediating role of Klemera–Doubal biological age acceleration (KDM-BAacc), while multiplicative and additive interaction analyses were conducted to assess effect modification by frailty. Sensitivity analyses were performed to evaluate the robustness of the findings.

**Results:**

During follow-up, 1,901 participants developed probable sarcopenia and 277 developed sarcopenia. Compared with the lowest ASI tertile, participants in the highest tertile had higher risks of probable sarcopenia (HR 1.38, 95% CI 1.22–1.55) and sarcopenia (HR 1.56, 95% CI 1.11–2.18). ASI showed a nonlinear association with probable sarcopenia but a linear association with sarcopenia. KDM-BAacc significantly mediated 8.66 and 18.85% of the associations with probable sarcopenia and sarcopenia, respectively. Frailty significantly modified these associations, with the associations largely confined to non-frail participants. These findings were robust in sensitivity analyses.

**Conclusion:**

Higher ASI was associated with increased risks of incident probable sarcopenia and sarcopenia. Biological aging partially mediated these associations, whereas frailty modified their magnitude. These findings identify arterial stiffness as a potential upstream determinant of sarcopenia, with biological aging as a mediating pathway and frailty as a modifier of susceptibility.

## 1 Introduction

Sarcopenia is an age-related syndrome characterized by progressive loss of skeletal muscle mass, strength, and function, substantially increasing risks of falls, disability, and mortality in older adults (1). The Asian Working Group for Sarcopenia (AWGS) has proposed the concept of probable sarcopenia, defined by low muscle strength or poor physical performance, providing an early warning stage for sarcopenia development (2). Early identification of its risk factors is therefore critical for healthy aging and public health.

Accumulating evidence suggests that sarcopenia is associated with chronic inflammation, mitochondrial dysfunction, and impaired protein homeostasis (3, 4), yet the upstream and shared determinants of these processes remain incompletely understood. Vascular aging, particularly arterial stiffening, has been proposed as one potential contributor to muscle decline through hemodynamic, microvascular, and inflammatory pathways (5, 6).

Arterial stiffness is a hallmark of vascular aging and is strongly associated with cardiovascular disease and all-cause mortality (7, 8). The arterial stiffness index (ASI), derived from photoplethysmography, is a non-invasive measure of arterial elasticity (9). Elevated ASI has been linked to increased risks of cardiovascular disease, diabetes, and cognitive impairment (10–12). Mechanistically, arterial stiffness may impair microcirculation and endothelial function, potentially promoting chronic inflammation, oxidative stress, and immune dysregulation (13–15). These processes may in turn affect pathways regulating muscle protein synthesis and degradation and thereby contribute to sarcopenia pathogenesis (3, 4, 16). Prior studies have reported positive associations between arterial stiffness–related indices and sarcopenia (5, 17). However, most evidence is cross-sectional, with substantial heterogeneity in sarcopenia definitions. In particular, the dose–response relationship between ASI and incident probable sarcopenia and sarcopenia remains unclear.

Biological aging reflects multisystem physiological decline and serves as a key link between aging-related diseases. Klemera–Doubal method biological age (KDM BA), derived from clinical biomarkers, captures systemic physiological deterioration and predicts multiple adverse health outcomes (18, 19). Accelerated KDM BA has been associated with both vascular aging and skeletal muscle decline (20, 21). Mechanistically, vascular aging may accelerate systemic aging via inflammatory and oxidative stress pathways (22), while biological age acceleration is closely linked to reduced muscle mass and function (21). Thus, biological aging may mediate the link between vascular aging and muscle deterioration, although large-scale cohort evidence remains limited.

Frailty is a vulnerability phenotype characterized by reduced physiological reserve and impaired stress response capacity, and is operationalized using the frailty index (FI), which quantifies cumulative health deficits (23). Unlike KDM BA, which reflects biological aging processes, FI emphasizes multisystem functional impairment and physiological reserve status at the clinical phenotypic level (24). Emerging evidence suggests that frailty status may influence individual susceptibility (25). However, its modifying effect on the association between ASI and sarcopenia remains unclear.

Using data from the UK Biobank, this study systematically examined the association between ASI and incident probable sarcopenia and sarcopenia. We further integrated vascular aging, biological aging, and frailty to evaluate the mediating role of KDM BA acceleration and the effect modification by FI, thereby clarifying the pathways linking vascular stiffening to muscle decline and the susceptibility underlying this association.

## 2 Methods

### 2.1 Study population

This study utilized data from a prospective cohort study based on the UK Biobank. This large-scale population-based cohort study recruited over 500,000 middle-aged and older adults across 22 assessment centers in England, Scotland, and Wales. Between 2006 and 2010, baseline data were collected, including biological samples, touchscreen questionnaires, and physical examinations (26). Ethical approval was granted by the North West Multi-Centre Research Ethics Committee (approval number: 11/NW/0382), and written informed consent was obtained from all participants. The present study was conducted under UK Biobank application number 1222281, in strict compliance with its data access policies and ethical guidelines.

The present study initially enrolled 501,938 participants. After excluding those lost to follow-up, who withdrew consent, or had missing baseline sarcopenia data, 489,455 participants free of probable sarcopenia and sarcopenia at baseline remained. Following further exclusion of those with missing follow-up sarcopenia data or missing ASI measurements, 32,451 participants were ultimately included in the analysis (Supplementary Figure S1).

### 2.2 Assessment of arterial stiffness index

The arterial stiffness index (ASI) was evaluated in 169,829 UK Biobank participants (2009–2010) with the PulseTrace PCA2, a finger photoplethysmography device. The time interval between the systolic and second diastolic peaks, known as the peak-to-peak time (PPT), corresponds to the pulse wave transit time to the lower body (27, 28). If the recorded waveform was inadequate, the reading was repeated on a larger finger or the thumb. Standing height was obtained using a Seca 202 stadiometer and entered manually. The index was then derived by dividing height (in meters) by PPT (in seconds). This operator-independent approach has been validated against carotid-femoral pulse wave velocity (29).

### 2.3 Assessment of biological aging

In this study, biological aging was assessed using the KDM BA algorithm, computed according to the method reported in previous studies (30, 31), with all parameters re-estimated within the current analytical sample. Sex-stratified models were trained on nine biomarkers measured at the same study visit among participants aged 30–75 years, detailed information is provided in Supplementary Table S1. For each sex, each biomarker was first regressed on chronological age, and the resulting regression parameters were then combined into a composite biological age score, as detailed in Supplementary Table S2. The algorithm allowed up to two missing biomarkers; participants with three or more missing values were excluded from score derivation. Based on the estimated KDM BA, we derived age acceleration (KDM-BAacc), defined as the residual from the regression of KDM BA on chronological age within the analytical sample. All computational routines were locally refitted, and no previously published frozen coefficients were used.

### 2.4 Assessment of frailty

Frailty was assessed using a 49-item Frailty Index (FI) based on the deficit accumulation model developed for UK Biobank participants (32, 33). Detailed definitions of all deficits and corresponding UK Biobank fields are provided in Supplementary Table S3. Each deficit was scored between 0 and 1 according to predefined criteria, with binary variables coded as 0 or 1 and ordinal variables assigned intermediate scores reflecting increasing severity. Disease ascertainment primarily relied on disease-specific UK Biobank fields and was supplemented by self-reported non-cancer illness codes, where appropriate. Hypercholesterolemia was identified using baseline lipid-lowering medication records. The FI was calculated as the sum of deficit scores divided by the number of non-missing deficits. Participants with fewer than 40 measurable deficits were excluded. Participants were categorized as non-frailty (FI < 0.25) and frailty (FI ≥ 0.25).

### 2.5 Ascertainment of outcomes

The study outcomes were probable sarcopenia and sarcopenia, defined in accordance with the 2019 criteria of the European Working Group on Sarcopenia in Older People 2 (EWGSOP2, 1) and adapted from previous UK Biobank-based investigations (34, 35). Sarcopenia was defined as the concomitant presence of (1) low handgrip strength (HGS), determined as the maximal value from both hands using a standardized protocol (thresholds: <27 kg in men, <16 kg in women), and (2) low muscle mass, assessed via bioelectrical impedance analysis-derived appendicular lean soft tissue (ALST), corrected according to previously described methods and adjusted for body mass index (BMI), with ALST/BMI thresholds of 0.84 for men and 0.55 for women (34). Probable sarcopenia was defined as low HGS alone. Incident events were ascertained through repeated HGS and ALST measurements across a maximum of four follow-up assessments, with the date of the first occurrence of probable sarcopenia or sarcopenia recorded as the event date. Follow-up time was calculated from the baseline date to the earliest of the first incident outcome, death, or administrative censoring.

### 2.6 Assessment of covariates

Participants self-reported their age, sex (female/male), ethnicity (White, Mixed, Asian/Asian British, Black/Black British, Chinese, or Other), and educational attainment (university/college degree, other qualifications, or none). The Townsend Deprivation Index was assigned using postal codes, with higher scores reflecting greater deprivation. Smoking status was categorized as never, former, or current, while alcohol intake was classified as ≥3 times/week, <3 times/week, or not current. Taking into account the influence of long-term chronic conditions, we included the number of long-term chronic conditions as a covariate, totaling 42 conditions, with detailed information provided in Supplementary Table S4.

### 2.7 Statistical analysis

Given our analytical objective of estimating the association between ASI and sarcopenia while minimizing potential confounding, we constructed a directed acyclic graph (DAG) using the DAGitty web tool (https://www.dagitty.net) to explicitly articulate our assumptions and guide covariate selection (36, 37). In this *a priori* DAG, ASI was specified as the exposure, incident (probable) sarcopenia as the outcome, KDM-BAacc as a mediator, and frailty as an effect modifier. By adjusting for a prespecified set of confounders to block all backdoor paths, we sought to reduce confounding bias in the estimated associations. The selection and definition of all covariates included in the adjustment set, along with the rationale for excluding KDM-BAacc and frailty from adjustment, are detailed in Supplementary Figure S2. The statistical models were selected according to the nature of the outcomes and prespecified analytical objectives.

Baseline characteristics are presented as means ± standard deviations (SDs) for continuous variables and as numbers (percentages) for categorical variables. Missing covariate data were handled using multiple imputation by chained equations (MICE) with five imputations. Cause-specific Cox proportional hazards models were used to evaluate the associations of ASI with incident probable sarcopenia and sarcopenia, given the time-to-event nature of the outcomes. ASI was prespecified to be analyzed both as a continuous variable and as tertiles. For tertile analyses, *P* for trend was calculated by modeling tertile categories as an ordinal variable. Three progressively adjusted models were fitted based on the prespecified covariate adjustment set informed by the *a priori* DAG. Model 1 was adjusted for age and sex. Model 2 was additionally adjusted for ethnicity, smoking status, and alcohol consumption. Model 3 was further adjusted for educational level, Townsend deprivation index, and the number of long-term conditions. Hazard ratios (HRs) and 95% confidence intervals (CIs) were reported. The proportional hazards assumption was evaluated using Schoenfeld residuals. Restricted cubic spline (RCS) analyses based on Model 3 were performed to examine potential nonlinear dose–response relationships beyond the assumption of linearity. Nonlinearity was assessed using likelihood ratio tests comparing models with and without spline terms. When significant nonlinearity was detected, two-piecewise Cox regression models were used to estimate the threshold effect.

Counterfactual mediation analysis was performed using the CMAverse package with Cox proportional hazards regression as the outcome model to evaluate the mediating role of KDM-BAacc. Total, natural direct, and natural indirect effects, together with the proportion mediated, were estimated after adjustment for Model 3 covariates. Exposure–mediator interaction was examined before mediation analysis, and no statistically significant interaction was identified.

To evaluate effect modification by frailty, analyses were stratified according to frailty status. Associations between ASI and the study outcomes were estimated separately within each subgroup using Model 3. RCS analyses were further performed within each frailty subgroup to compare the dose–response relationships. Multiplicative interaction was assessed by including a product term between ASI and frailty status in the Cox models. To evaluate additive interaction, ASI was dichotomized at the median into low- and high-ASI groups and jointly classified with frailty status into four exposure categories. Relative excess risk due to interaction (RERI) and its corresponding 95% CI were calculated to quantify interaction on the additive scale.

Several sensitivity analyses were performed. First, Fine–Gray subdistribution hazards models accounting for death as a competing event were used to repeat both the primary association analyses and the interaction analyses. Second, complete-case analyses excluding participants with missing covariate data were conducted. Finally, stratified analyses were performed according to age group, sex, hypertension, coronary heart disease, dislipidemia, heart failure, depression, connective tissue disease, osteoporosis, dementia, COPD, fractures, and falls in last year to assess the consistency of the associations across clinically relevant subgroups.

All statistical analyses were performed using R software (version 4.4.1; R Foundation for Statistical Computing, Vienna, Austria). A two-sided *p* value <0.05 was considered statistically significant.

## 3 Results

### 3.1 Participant characteristics

Baseline characteristics stratified by ASI tertiles are presented in Table 1. The mean age of the participants was 55.1 ± 7.6 years, and 51.7% were women. During follow-up, a total of 1,901 and 277 participants developed incident probable sarcopenia and sarcopenia, respectively. Compared with those in the lowest ASI tertile, participants with higher ASI tended to be older, had a greater burden of long-term conditions, exhibited more advanced biological aging, and showed a higher degree of frailty.

**Table 1:** Baseline characteristics of the study population according to arterial stiffness index tertiles.

Participant characteristics	Total	Arterial stiffness index			*p* value
		Tertile 1	Tertile 2	Tertile 3	
No. of participants	32,451	10,817	10,812	10,822	
Age, years	55.1 ± 7.6	53.0 ± 7.8	54.7 ± 7.5	57.5 ± 6.8	<0.001
Gender, *n* (%)					<0.001
Female	16,786 (51.7)	7,245 (67)	5,288 (48.9)	4,253 (39.3)	
Male	15,665 (48.3)	3,572 (33)	5,524 (51.1)	6,569 (60.7)	
Ethnic groups, *n* (%)					<0.001
White	30,945 (95.4)	10,249 (94.7)	10,275 (95)	10,421 (96.3)	
Mix	208 (0.6)	82 (0.8)	66 (0.6)	60 (0.6)	
Asian or Asian British	528 (1.6)	181 (1.7)	192 (1.8)	155 (1.4)	
Black or Black British	408 (1.3)	159 (1.5)	151 (1.4)	98 (0.9)	
Chinese and others	362 (1.1)	146 (1.3)	128 (1.2)	88 (0.8)	
Drinking status, *n* (%)					<0.001
≥ 3 times per week	16,460 (50.7)	5,189 (48)	5,410 (50)	5,861 (54.2)	
< 3 times per week	11,722 (36.1)	4,100 (37.9)	3,960 (36.6)	3,662 (33.8)	
Not current	4,269 (13.2)	1,528 (14.1)	1,442 (13.3)	1,299 (12)	
Smoking status, *n* (%)					<0.001
Non-smoker	19,408 (59.8)	7,021 (64.9)	6,504 (60.2)	5,883 (54.4)	
Former smoker	10,991 (33.9)	3,262 (30.2)	3,596 (33.3)	4,133 (38.2)	
Current smoker	2,052 (6.3)	534 (4.9)	712 (6.6)	806 (7.4)	
Educational level, *n* (%)					<0.001
College or university degree	15,574 (48.0)	5,447 (50.4)	5,330 (49.3)	4,797 (44.3)	
Any other qualification	15,084 (46.5)	4,906 (45.4)	4,924 (45.5)	5,254 (48.5)	
No qualification	1,793 (5.5)	464 (4.3)	558 (5.2)	771 (7.1)	
Townsend deprivation index	−2.4 (−3.7, −0.2)	−2.3 (−3.6, −0.1)	−2.3 (−3.7, 0.0)	−2.5 (−3.8, −0.4)	<0.001
No. of long-term conditions, *n* (%)					<0.001
0	13,650 (42.1)	5,065 (46.8)	4,624 (42.8)	3,961 (36.6)	
1	10,990 (33.9)	3,575 (33)	3,571 (33)	3,844 (35.5)	
3	5,127 (15.8)	1,465 (13.5)	1,727 (16)	1,935 (17.9)	
4	1,850 (5.7)	499 (4.6)	600 (5.5)	751 (6.9)	
5	600 (1.8)	162 (1.5)	199 (1.8)	239 (2.2)	
>5	234 (0.7)	51 (0.5)	91 (0.8)	92 (0.9)	
Arterial Stiffness Index, m/s	9.0 ± 2.9	6.1 ± 0.9	8.7 ± 0.8	12.3 ± 2.0	<0.001
KDM-BAacc, years	−2.2 (−7.8, 3.3)	−2.5 (−7.9, 2.8)	−2.4 (−8.0, 3.2)	−1.7 (−7.4, 3.9)	<0.001
Frailty index	0.11 ± 0.06	0.11 ± 0.06	0.12 ± 0.06	0.12 ± 0.06	<0.001
Frailty status, *n* (%)					<0.001
Non-frailty	30,920 (95.28)	10,377 (95.93)	10,263 (94.92)	10,280 (94.99)	
Frailty	1,531 (4.72)	440 (4.07)	549 (5.08)	542 (5.01)	
Probable sarcopenia, *n* (%)	1,901 (5.86)	514 (4.75)	614 (5.68)	773 (7.14)	<0.001
Sarcopenia, *n* (%)	277 (0.85)	49 (0.45)	90 (0.83)	138 (1.28)	<0.001

Continuous variables are presented as mean ± standard deviation and median (interquartile range), and categorical variables as counts (percentages). KDM-BAacc, Klemera–Doubal biological age acceleration. The data after using multiple imputation.

### 3.2 Associations between arterial stiffness index and the risks of probable sarcopenia and sarcopenia

Table 2 presents the associations between ASI and the risks of incident probable sarcopenia and sarcopenia. In the fully adjusted model, each 1 m/s increase in ASI was associated with a 3% higher risk of probable sarcopenia (*p* < 0.001) and a 5% higher risk of sarcopenia (*p* = 0.015). In comparison, each additional year of chronological age was associated with an 8 and 12% higher hazard of probable sarcopenia (HR, 1.08; 95% CI, 1.07–1.09) and sarcopenia (HR, 1.12; 95% CI, 1.10–1.15), respectively. When ASI was categorized into tertiles, participants in the highest tertile had significantly higher risks of both probable sarcopenia (HR, 1.38; 95% CI, 1.22–1.55) and sarcopenia (HR, 1.56; 95% CI, 1.11–2.18) than those in the lowest tertile after full adjustment.

**Table 2:** Associations between arterial stiffness index and the risks of probable sarcopenia and sarcopenia.

Variable (events/total)	Model 1		Model 2		Model 3	
	HR (95% CI)	*p* value	HR (95% CI)	*p* value	HR (95% CI)	*p* value
Probable sarcopenia						
ASI increase per 1 m/s	1.04 (1.02, 1.05)	<0.001	1.04 (1.02, 1.05)	<0.001	1.03 (1.02, 1.05)	<0.001
Tertile 1 (514/10,817)	1(Ref)		1(Ref)		1(Ref)	
Tertile 2 (614/10,812)	1.29 (1.14, 1.45)	<0.001	1.29 (1.15, 1.46)	<0.001	1.28 (1.14, 1.44)	<0.001
Tertile 3 (773/10,822)	1.39 (1.24, 1.57)	<0.001	1.41 (1.25, 1.58)	<0.001	1.38 (1.22, 1.55)	<0.001
*p* for trend		<0.001		<0.001		<0.001
Sarcopenia						
ASI increase per 1 m/s	1.05 (1.01, 1.10)	0.007	1.05 (1.01, 1.10)	0.007	1.05 (1.01, 1.09)	0.015
Tertile 1 (49/10,817)	1(Ref)		1(Ref)		1(Ref)	
Tertile 2 (90/10,812)	1.55 (1.09, 2.20)	0.014	1.55 (1.09, 2.20)	0.014	1.48 (1.04, 2.11)	0.028
Tertile 3 (138/10,822)	1.62 (1.16, 2.27)	0.005	1.63 (1.16, 2.27)	0.005	1.56 (1.11, 2.18)	0.01
*p* for trend		0.008		0.008		0.016

Model 1 was adjusted for age and sex; Model 2 was adjusted for ethnic groups, drinking status, and smoking status; Model 3 was further adjusted for educational level, Townsend deprivation index, and number of long-term conditions. ASI, arterial stiffness index; HR, hazard ratio; CI, confidence interval; Ref, reference. Hazard ratios were estimated using multivariable Cox proportional hazards models.

RCS analyses further demonstrated distinct dose–response patterns for the two outcomes (Figures 1A,B). A nonlinear association was observed between ASI and probable sarcopenia (*P* for nonlinearity = 0.029), with an apparent threshold at approximately 13.5 m/s (Supplementary Table S5). The risk of probable sarcopenia increased with increasing ASI below this threshold but reached a plateau thereafter. In contrast, the association between ASI and sarcopenia was approximately linear across the observed range of ASI, with no evidence of nonlinearity.

**Figure 1:**
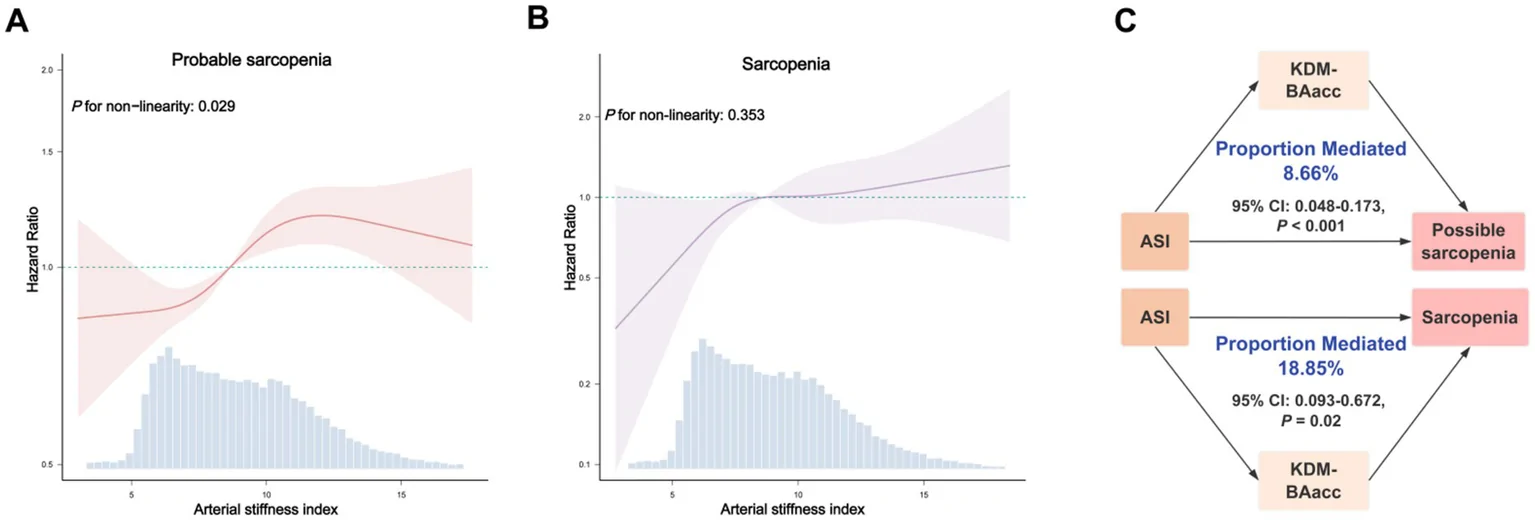
Restricted cubic spline analyses and mediation analysis of the associations of arterial stiffness index with probable sarcopenia and sarcopenia. **(A)** Restricted cubic spline showing the association between arterial stiffness index (ASI) and incident probable sarcopenia. **(B)** Restricted cubic spline showing the association between arterial stiffness index (ASI) and incident sarcopenia. **(C)** Mediation diagram illustrating the role of Klemera–Doubal method biological age acceleration (KDM-BAacc) in the associations between ASI and incident probable sarcopenia and sarcopenia. Solid lines represent hazard ratios (HRs), and shaded areas represent 95% confidence intervals (95% CIs).

### 3.3 Mediating effect of biological age acceleration

Counterfactual mediation analysis demonstrated that KDM-BAacc significantly mediated the associations between ASI and both probable sarcopenia and sarcopenia (Figure 1C; Supplementary Tables S6, S7). For probable sarcopenia, both the natural direct effect and the natural indirect effect mediated through KDM-BAacc were statistically significant, with an estimated mediation proportion of 8.66% (95% CI, 4.81–17.34%). Similarly, KDM-BAacc significantly mediated the association between ASI and incident sarcopenia. KDM-BAacc accounted for 18.85% (95% CI, 9.32–67.22%) of the association between ASI and sarcopenia.

### 3.4 Effect modification by frailty status

The numbers and percentages of incident probable sarcopenia and sarcopenia cases according to frailty status are shown in Supplementary Table S8. The associations between ASI and incident probable sarcopenia and sarcopenia differed according to frailty status (Figure 2). In non-frail participants, per 1 m/s increase in ASI, HRs were 1.04 (95% CI, 1.02–1.05) for probable sarcopenia and 1.06 (1.02–1.10) for sarcopenia; no significant associations were found in frail participants. Multiplicative interaction was significant for sarcopenia (*p* = 0.013) and borderline for probable sarcopenia. When ASI was categorized into tertiles, interactions were significant for both probable sarcopenia (*p* = 0.031) and sarcopenia (*p* = 0.022).

**Figure 2:**
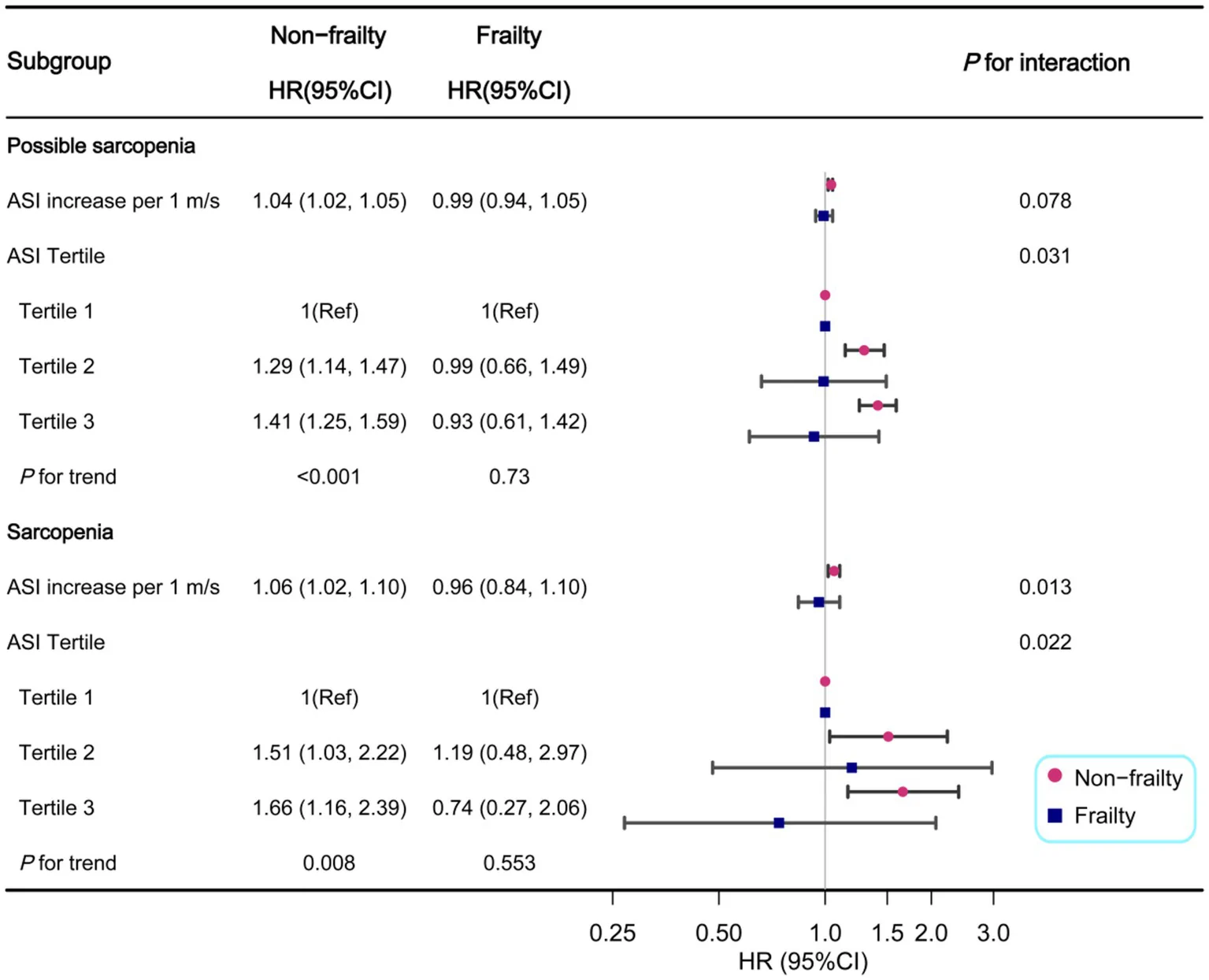
Stratified associations of arterial stiffness index with incident probable sarcopenia and sarcopenia according to frailty status. Forest plots showing the associations between arterial stiffness index (ASI) and incident probable sarcopenia and sarcopenia among non-frail and frail participants. ASI was modeled as both a continuous variable and tertiles. Hazard ratios (HRs) and 95% confidence intervals (95% CIs) were derived from multivariable-adjusted Cox proportional hazards models. Adjusted for age, sex, ethnic group, drinking status, and smoking status, educational level, Townsend deprivation index, and number of long-term conditions. *p* values for interaction indicate whether the associations between ASI and the outcomes differed according to frailty status.

Joint effect analysis suggested a potential interaction (Figures 3A,B). Compared with non-frail participants with low ASI (reference group), the risk of probable sarcopenia was elevated in non-frail participants with high ASI (HR = 1.34) and in frail participants with low ASI (HR = 1.77). However, the joint exposure of frailty and high ASI did not show a further increase in risk (HR, 1.63; 95% CI, 1.28–2.08). Multiplicative interaction was observed (HR for interaction = 0.69; 95% CI, 0.49–0.96), and the RERI was −0.48 (95% CI, −1.05 to 0.08).

**Figure 3:**
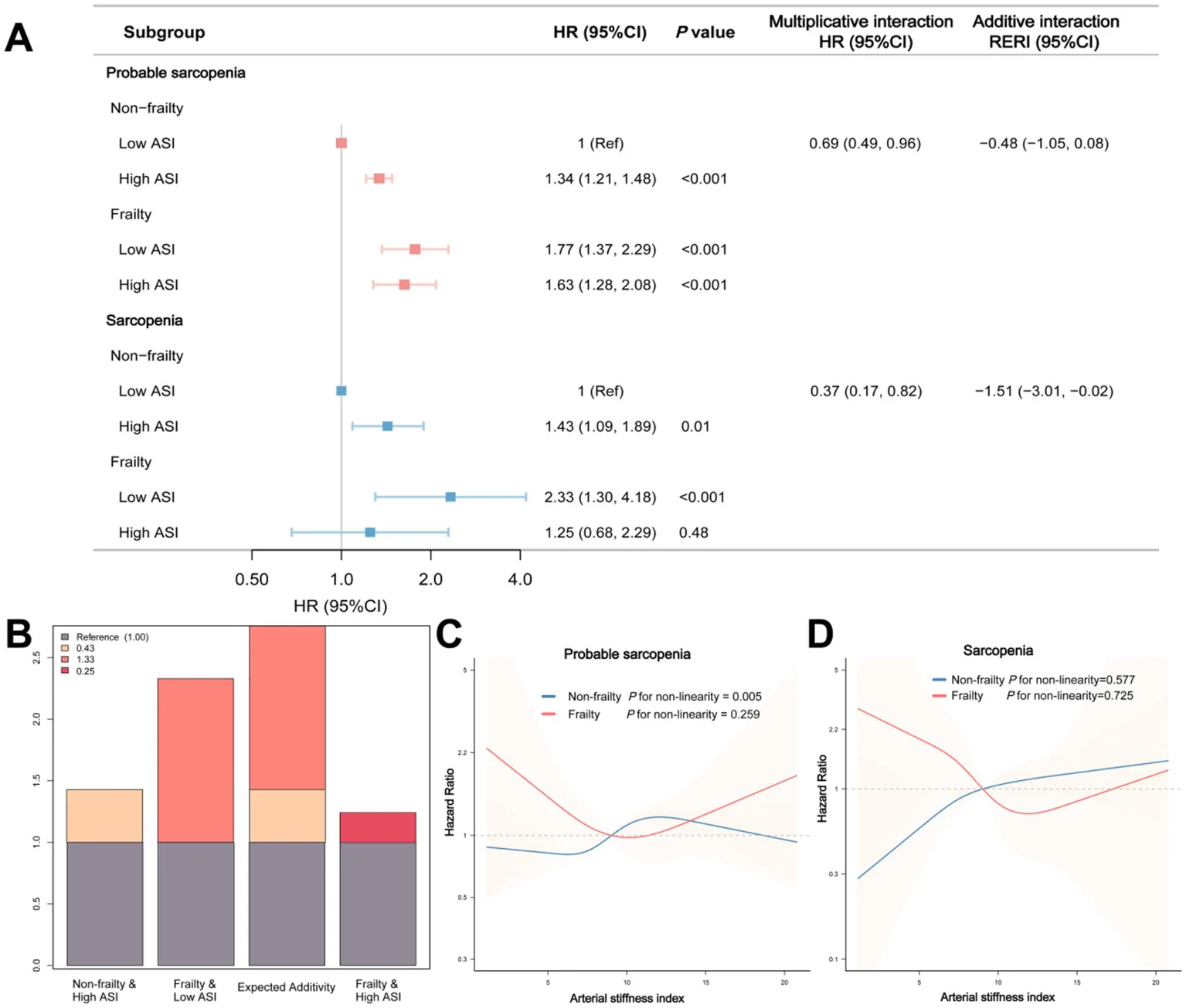
Interaction analyses of arterial stiffness index and frailty status in relation to incident probable sarcopenia and sarcopenia. **(A)** Joint associations of arterial stiffness index (ASI) and frailty status with incident probable sarcopenia and sarcopenia. Participants were categorized into four groups according to frailty status (non-frailty or frailty) and ASI level (low or high). Hazard ratios (*HRs*) and 95% confidence intervals (95% *CIs*) were estimated using multivariable-adjusted Cox proportional hazards models. Measures of interaction on both multiplicative and additive scales are presented, including the interaction term *HR* and the relative excess risk due to interaction (*RERI*). **(B)** Additive interaction analysis for incident sarcopenia, illustrating the joint effects of ASI and frailty status on the absolute excess risk scale. **(C)** Restricted cubic spline analyses showing the association between ASI and incident probable sarcopenia according to frailty status. **(D)** Restricted cubic spline analyses showing the association between ASI and incident sarcopenia according to frailty status. All models were adjusted for age, sex, ethnic group, drinking status, and smoking status, educational level, Townsend deprivation index, and number of long-term conditions. Shaded areas represent 95% confidence intervals.

For sarcopenia, a similar joint pattern was observed. Non-frail participants with high ASI showed increased risk (HR = 1.43), whereas frail participants with low ASI exhibited the highest risk (HR = 2.33). In contrast, frail participants with high ASI did not show a statistically significant association (HR, 1.25; 95% CI, 0.68–2.29). Multiplicative interaction was significant (HR for interaction = 0.37; 95% CI, 0.17–0.82), and additive interaction analysis indicated a negative departure from additivity (RERI = −1.51; 95% CI, −3.01 to −0.02).

RCS analyses further illustrated heterogeneity in dose–response relationships by frailty status (Figures 3C,D). For probable sarcopenia, a significant nonlinear association was observed in non-frail participants, with an inflection point at approximately 13.5 m/s (Supplementary Table S9). In contrast, no significant nonlinear association was observed among frail participants. For sarcopenia, no significant nonlinearity was detected in either subgroup; however, the direction of associations differed between frail and non-frail participants, indicating a clear effect modification by frailty status.

### 3.5 Sensitivity analyses

Sensitivity analyses showed robust results. Fine–Gray competing risk models produced similar estimates to the main cause-specific Cox models for both outcomes and interaction analyses (Supplementary Table S10; Supplementary Figure S3). Complete-case analyses yielded comparable results to the primary analyses (Supplementary Table S11). Stratified analyses by age group, sex, hypertension, coronary heart disease, dislipidemia, heart failure, depression, connective tissue disease, osteoporosis, dementia, COPD, fractures, and falls in last year did not reveal significant interactions (Supplementary Tables S12, S13).

## 4 Discussion

In this large prospective cohort study of 32,451 participants, higher arterial stiffness index was independently associated with increased risks of incident probable sarcopenia and sarcopenia. The association with probable sarcopenia was nonlinear, whereas a linear relationship was observed for sarcopenia. KDM-BAacc significantly mediated these associations, accounting for 8.66 and 18.85% of the effects on probable sarcopenia and sarcopenia, respectively. Importantly, frailty status substantially modified these associations, with associations evident in non-frail participants but markedly attenuated or absent in frail individuals, accompanied by significant multiplicative and additive interactions, particularly for sarcopenia. These findings suggest that higher arterial stiffness is associated with subsequent muscle decline and that biological aging may partially account for this association, while the magnitude of the association varies according to frailty status.

Our findings are consistent with previous studies reporting positive associations between arterial stiffness and sarcopenia-related outcomes (6, 38, 39). ASI showed a linear association with sarcopenia, whereas a plateauing relationship was observed for probable sarcopenia at higher ASI levels. Similar nonlinear associations have been reported previously (5). One possible explanation is that probable sarcopenia represents an early functional stage defined primarily by grip strength, which may be more sensitive to early vascular dysfunction than muscle mass. Accordingly, modest increases in ASI may be associated with a relatively rapid increase in risk, whereas beyond a certain level, further increases in ASI may confer diminishing incremental risk, resulting in a plateauing pattern. In contrast, sarcopenia reflects more established structural muscle loss that may accumulate over prolonged periods of vascular and metabolic dysfunction (40, 41), potentially resulting in a more sustained association with ASI. These differences should nevertheless be interpreted cautiously, as the observed dose–response patterns do not by themselves establish distinct biological mechanisms.

The biological basis for a potential directional relationship between arterial stiffness and subsequent muscle deterioration warrants careful consideration. Importantly, chronic inflammation, oxidative stress, and biological aging are shared processes that may contribute to both arterial stiffening and skeletal muscle decline; therefore, these pathways alone cannot distinguish arterial stiffness as a cause from a concurrent manifestation of systemic aging. A potentially directional pathway may instead arise from the downstream hemodynamic consequences of established arterial stiffening. Increased arterial stiffness reduces arterial compliance and enhances pulse-wave reflection, thereby increasing pulsatile load and potentially impairing peripheral tissue perfusion (42). Persistent alterations in vascular hemodynamics may impair microvascular function, reduce oxygen and nutrient delivery to skeletal muscle, and contribute to endothelial dysfunction and capillary rarefaction (13). These vascular abnormalities may subsequently affect skeletal muscle homeostasis by limiting anabolic signaling, mitochondrial function, and tissue regenerative capacity (16, 43–47). In this context, vascular stiffness may plausibly contribute to muscle deterioration through hemodynamic and microvascular pathways that occur downstream of the structural vascular abnormality, rather than solely through inflammatory or oxidative mechanisms that are shared across aging-related phenotypes. Nevertheless, this proposed direction should not be interpreted as evidence of a unidirectional causal pathway. Thus, our findings are compatible with both a model in which pre-existing arterial stiffness contributes to subsequent muscle decline and a shared-pathology model in which vascular and skeletal muscle aging arise partly from common upstream processes. The prospective design establishes temporal precedence of ASI over the ascertainment of incident sarcopenia, which strengthens the plausibility of the former interpretation, but does not distinguish these causal models definitively.

We also observed that KDM-BAacc statistically accounted for part of the associations between ASI and both outcomes, suggesting that systemic biological aging may represent one pathway linking vascular aging and subsequent muscle decline. ASI was selected because it captures a specific dimension of vascular aging, namely arterial elasticity, whereas KDM-BAacc reflects systemic biological aging based on a composite of multiple biomarkers. Thus, these two measures were considered complementary rather than interchangeable indicators of aging. Arterial stiffness may be linked to systemic biological aging through chronic vascular stress, oxidative imbalance, and impaired microcirculatory function (16). Conversely, biological aging itself may promote vascular stiffening and skeletal muscle deterioration through shared mechanisms such as cellular senescence, impaired tissue repair, and disrupted proteostasis (48). Importantly, the persistence of a statistically significant direct association after accounting for KDM-BAacc suggests that the association between ASI and sarcopenia was not fully explained by this particular composite measure of biological age. However, this finding does not establish that ASI is independent of biological aging more broadly, as other biological-age measures may capture overlapping or complementary dimensions of aging. Overall, the observed mediation pattern is consistent with a model in which vascular aging may be linked to sarcopenia partly through systemic biological aging pathways. Nevertheless, given the observational nature of the data, these findings do not establish that vascular aging causally accelerates biological aging or that biological aging causally mediates the association between ASI and sarcopenia.

Frailty significantly modified the associations between ASI and incident probable sarcopenia and sarcopenia, with positive associations observed primarily among non-frail participants. Although frailty and sarcopenia are closely related, they represent distinct constructs: sarcopenia primarily characterizes impaired muscle strength and muscle mass, whereas the frailty index captures cumulative deficits across multiple physiological systems and reflects broader multisystem vulnerability and reduced physiological reserve. Therefore, examining frailty as an effect modifier provides information beyond the occurrence of sarcopenia itself by addressing whether the association between vascular stiffness and subsequent muscle decline varies according to overall physiological reserve. In non-frail individuals, relatively preserved physiological reserve may make the contribution of vascular dysfunction to subsequent muscle deterioration more apparent (49, 50). In contrast, among frail individuals with substantial multisystem impairment and high baseline vulnerability, the relative contribution of any single vascular factor may be attenuated, potentially explaining the weaker observed association. This interpretation is consistent with the observed effect modification but remains hypothesis-generating and cannot establish the underlying biological mechanism.

Finally, from an epistemological standpoint, we note that the analyses presented here are explanatory rather than predictive. Explanatory modeling aims to estimate and test associations and the underlying mechanisms, whereas predictive modeling aims to forecast outcomes in new individuals (51). Our DAG-based causal framework, the use of cause-specific Cox models, mediation analysis, and interaction analysis, and the terminology of “association,” “mediation,” and “effect modification” all reflect an explanatory goal. Accordingly, although our findings suggest that arterial stiffness may act as an upstream factor in muscle decline and that frailty delineates differential susceptibility, we do not claim to provide a validated predictive or screening tool; any future translational use for prospective risk stratification would require independent external validation in dedicated prediction studies.

This study has several strengths, including its prospective design, large sample size, repeated follow-up, and a comprehensive investigation of the associations of arterial stiffness with probable sarcopenia and sarcopenia through the integration of biological aging mediation analysis and frailty interaction analysis. Several limitations of this study should be acknowledged. First, as an observational study, causal inference cannot be established. Although ASI was measured before the occurrence of incident sarcopenia, providing temporal precedence, this temporal ordering alone does not establish causality. In particular, chronic inflammation, oxidative stress, and biological aging may represent shared upstream processes contributing to both arterial stiffness and muscle deterioration. Therefore, residual confounding, shared causation, and alternative causal structures cannot be completely excluded despite adjustment for measured covariates and the use of an *a priori* DAG. The mediation findings should likewise be interpreted as evidence of statistical mediation rather than definitive proof of a causal biological pathway. Second, sarcopenia was not continuously monitored during follow-up, which may have introduced a certain degree of outcome misclassification. Third, the number of incident sarcopenia cases was relatively small, particularly among frail participants, which may have limited the statistical power of subgroup and interaction analyses and resulted in imprecise estimates. Therefore, the observed differences in dose–response patterns between frailty groups should be interpreted cautiously and require confirmation in future studies with larger numbers of incident cases. Fourth, we did not compare ASI with other biological or vascular aging measures. Although the association between ASI and sarcopenia was not fully explained by KDM-BAacc, as indicated by the residual direct association after accounting for KDM-BAacc, this does not establish the unique value of ASI beyond other aging-related biomarkers. Alternative measures, including PhenoAge and epigenetic clocks, were not examined and warrant evaluation in future studies. Fifth, although we adjusted for multiple socioeconomic, lifestyle, and health-related factors, residual confounding by other incompletely measured lifestyle characteristics, such as dietary patterns and broader health behaviors, cannot be completely excluded. Finally, UK Biobank participants are predominantly of white European ancestry, which may limit the generalizability of these findings to other populations.

## 5 Conclusion

In this large prospective cohort study, higher ASI was associated with increased risks of incident probable sarcopenia and sarcopenia, with distinct dose–response patterns across outcomes. Biological age acceleration partially mediated these associations, suggesting involvement of aging-related pathways in linking vascular stiffening to muscle decline. Importantly, frailty status significantly modified these associations, with attenuated or absent effects observed in frail participants and evidence of both multiplicative and additive interactions. These findings support arterial stiffness as a potential upstream factor in muscle deterioration and highlight heterogeneity in susceptibility according to frailty status, suggesting that vascular stiffening contributes to muscle decline partly through aging-related pathways and that this contribution varies by frailty.

## Data Availability

Publicly available datasets were analyzed in this study. This data can be found here: Data from the UK Biobank are available to researchers upon application at https://www.ukbiobank.ac.uk. The analytic code and supporting materials used in this study will be made available by the corresponding author upon reasonable request.
