# Supplementary material for: Biological aging mediates and frailty modifies the associations of arterial stiffness with incident probable sarcopenia and sarcopenia: a prospective cohort study of UK biobank
Source: Front Public Health. 2026 Sep 14;14:1926399. doi: 10.3389/fpubh.2026.1926399 (PMC13616727; doi:10.3389/fpubh.2026.1926399)
Supplement: Supplementary file 1 [file Table_1.DOCX]

**Figure S1.** The flowchart of the study.

**Figure S2.**  Directed Acyclic Graph (DAG) of the Hypothesized Variable Relationships.

Directed acyclic graph (DAG) illustrating the hypothesized relationships among ASI, incident (probable) sarcopenia, KDM‑BAacc, frailty, and confounders. ASI was specified as the exposure, incident (probable) sarcopenia as the outcome, and KDM‑BAacc as a mediator. Frailty was specified as an effect modifier of the ASI–sarcopenia association.

Covariates were selected a priori as common causes (confounders) of both arterial stiffness and sarcopenia, including age, sex, ethnicity, smoking status, alcohol intake, educational level, Townsend deprivation index, and number of long‑term conditions. Age and sex are the dominant determinants of both vascular stiffening and muscle decline[1, 2]; smoking and alcohol intake are established lifestyle determinants of arterial stiffness[3]; educational level and the Townsend deprivation index capture socioeconomic position, which is associated with reduced muscle mass, strength, and physical function[4]; and the number of long‑term conditions reflects multimorbidity burden, a recognized risk factor for incident sarcopenia[5]. KDM‑BAacc, a mediator, and frailty, an effect modifier, were deliberately excluded from the adjustment set to avoid overadjustment bias. KDM‑BAacc, Klemera–Doubal biological age acceleration; ASI, arterial stiffness index.

**Figure S3.** Stratified associations of arterial stiffness index with incident possible sarcopenia and sarcopenia according to frailty status in competing risk models.

Forest plots showing the associations between arterial stiffness index (ASI) and incident possible sarcopenia and sarcopenia among non-frail and frail participants. ASI was modeled as both a continuous variable and tertiles. Subdistribution hazard ratio (SHR) and 95% confidence interval (95% CI) were estimated using Fine–Gray models accounting for the competing risk of death. Adjusted for age, sex, ethnic group, drinking status, and smoking status, educational level, Townsend deprivation index, and number of long-term conditions. P values for interaction indicate whether the associations between ASI and the outcomes differed according to frailty status.

**Table S1.** Items used to compose the Klemera–Doubal biological age in included population

| **Item** | **Field ID** |
| --- | --- |
| Forced expiratory volume in 1-second (FEV1) | 3063 |
| Systolic blood pressure, automated reading | 4080 |
| Cholesterol | 30690 |
| Glycated hemoglobin (HbA1c) | 30750 |
| Albumin | 30600 |
| Creatinine | 30700 |
| C-reactive protein | 30710 |
| Alkaline phosphatase | 30610 |
| Urea | 30670 |
| Age | 21022 |

**Table S2.** The detailed calculation process of Klemera–Doubal biological age

| **Methods** | **Description** |
| --- | --- |
| **Biomarker pre-processing and units** | The nine biomarkers and their conversions were: FEV₁ (mean of two readings, mL); systolic blood pressure (automated reading preferred, manual as fallback, mean of two readings, mmHg); total cholesterol (mg/dL); HbA1c (%); albumin (g/dL); creatinine (mg/dL); C-reactive protein (entered as log[1 + CRP in mg/L]); alkaline phosphatase (U/L); urea (as BUN proxy, BUN mg/dL). Chronological age was taken from the assessment centre visit, with recruitment age used if missing. |
| **KDM model fitting (sex-specific)** | For each sex separately, and for each biomarker $i (i=1,\ldots,9i=1,\ldots,9)$, we fitted a simple linear regression:  $x_{i}=q_{i}+k_{i}*age+\varepsilon_{i}$  obtaining the intercept $qi$, slope $ki$, residual standard deviation $si$, and $R2=ri$,  From these we computed:  $r_{1}i=\vert k_{i}/(s_{i}r_{i})\vert,r_{2}i=\vert k_{i}/s_{i}\vert,n_{2}i=(k_{i}/s_{i})^{2}$  $r_{c}har=(\sum_{(}i=1)^{9}r_{1}i)/(\sum_{(}i=1)^{9}r_{2}i)$  $s_{r}=(1-r_{c}har^{2})/(r_{c}har^{2})*(age_{m}ax-age_{m}in)^{2}/(12*9)$  For each individual jj in the training set, a preliminary biological age was computed as:  ${BA}_{E,j}=\frac{\sum_{i} (x_{ij}-q_{i})\cdot\frac{k_{i}}{s_{i}^{2}}}{\sum_{i} n_{2i}}\times\frac{9}{n_{obs,j}},$  where $nobs,j$is the number of available biomarkers for that individual (those with >2 missing biomarkers excluded). The variance of $BAE,j-agej$across all training individuals, $s2$  was used to obtain:  $s_{ba}^{2}=s^{2}-s_{r}.$ |
| **Scoring of biological age** | For any individual in the scoring sample, the final KDM biological age was calculated with the sex-specific parameters:  ${KDMBA}_{j}=\frac{\sum_{i} \left[ (x_{ij}-q_{i})\cdot\frac{k_{i}}{s_{i}^{2}} \right]+\frac{{age}_{j}}{s_{ba}^{2}}}{\sum_{i} n_{2i}+\frac{1}{s_{ba}^{2}}}.$  Up to two biomarkers could be missing; individuals missing three or more biomarkers received no KDM BA. |
| **Derived variables** | **KDM BA difference:** ${KDMBA}_{diff}=KDMBA-chronologicalage.$  **KDM-BAacc: residuals from the linear regression** $KDMBA\sim chronologicalage$ fitted within the analytic sample. |

**Table S3.** Items used to compose the frailty index in included population

| **Deficit** | **Item** | **Trait** | **Field ID** | **Scoring method** | **Score of 1, n (%)** |
| --- | --- | --- | --- | --- | --- |
| Sensory | 1 | Glaucoma | 2227 | Yes = 1 No = 0 Missing if "prefer not to answer" for data-field 6148. | 392 (1.21) |
|  |  |  | 20002 |  |  |
|  | 2 | Cataracts | 6148 | Yes = 1 No = 0 Missing if "prefer not to answer" for data-field 6148. | 1014 (3.21) |
|  |  |  | 20002 |  |  |
|  | 3 | Hearing difficulty | 2247 | Yes and completely deaf = 1 No = 0 | 7817 (24.09) |
| Cranial | 4 | Migraine | 2473 | Yes = 1 No = 0 Missing if "prefer not to answer" for data-field 2473. | 819 (2.52) |
|  |  |  | 20002 |  |  |
|  | 5 | Dental problems | 6149 | None = 0 Any = 1 | 10964 (33.79) |
| Mental wellbeing | 6 | Self-rated health | 2178 | Excellent = 0 Good = 0.25 Fair = 0.5 Poor = 1 | 650 (2.00) |
|  | 7 | Fatigue: frequency of tiredness / lethargy in last two weeks | 2080 | Not at all = 0 Several days = 0.25 More than half = 0.5 Nearly every day = 1 | 1261 (3.89) |
|  | 8 | Sleep: experience of sleeplessness/insomnia | 1200 | Never/rarely = 0  Sometimes = 0.5 Usually = 1 | 8096 (24.95) |
|  | 9 | Depressed feelings: frequency in last two weeks | 2050 | Not at all = 0 Several days = 0.5 More than half = 0.75 Nearly every day = 1 | 361 (1.11) |
|  | 10 | Self-described nervous personality | 1970 | Yes = 1 No = 0 | 6086 (18.75) |
|  | 11 | Severe anxiety/ panic attacks | 2473 | Yes = 1 No = 0 Missing if "prefer not to answer" for data-field 2473. | 337 (1.04) |
|  |  |  | 20002 |  |  |
|  | 12 | Common to feel loneliness | 2020 | Yes = 1 No = 0 | 4583 (14.12) |
|  | 13 | Sense of misery (ever/never) | 1930 | Yes = 1 No = 0 | 12797 (39.43) |
| Infirmity | 14 | Infirmity: long-standing illness or disability | 2188 | Yes = 1 No = 0 | 7917 (24.4) |
|  | 15 | Falls in last year | 2296 | No falls = 0 Only one fall = 0.5 More than one fall = 1 | 3983 (12.27) |
|  | 16 | Fractures/broken bones in last five years | 2463 | Yes = 1 No = 0 | 2739 (8.44) |
| Cardiometabolic | 17 | Diabetes | 2443 | Yes = 1 No = 0 Missing if "prefer not to answer" for data-field 2443. | 974 (3.00) |
|  |  |  | 20002 |  |  |
|  | 18 | Myocardial infarction | 6150 | Yes = 1 No = 0 Missing if "prefer not to answer" for data-field 6150. | 383 (1.18) |
|  |  |  | 20002 |  |  |
|  | 19 | Angina | 6150 | Yes = 1 No = 0 Missing if "prefer not to answer" for data-field 6150. | 505 (1.56) |
|  |  |  | 20002 |  |  |
|  | 20 | Stroke | 6150 | Yes = 1 No = 0 Missing if "prefer not to answer" for data-field 6150. | 212 (0.65) |
|  |  |  | 20002 |  |  |
|  | 21 | High blood pressure | 6150 | Yes = 1 No = 0 Missing if "prefer not to answer" for data-field 6150. | 6561 (20.22) |
|  |  |  | 20002 |  |  |
|  | 22 | Hypothyroidism | 2473 | Yes = 1 No = 0 Missing if "prefer not to answer" for data-field 2473. | 1290 (3.98) |
|  |  |  | 20002 |  |  |
|  | 23 | Deep-vein thrombosis | 6152 | Yes = 1 No = 0 Missing if "prefer not to answer" for data-field 6152. | 428 (1.32) |
|  |  |  | 20002 |  |  |
|  | 24 | High cholesterol | 6153 | Yes = 1 No = 0 Missing if "prefer not to answer" for data-field 6153 or 6177. | 4056 (12.5) |
|  |  |  | 6177 |  |  |
| Respiratory | 25 | Breathing: wheeze in last year | 2316 | Yes = 1 No = 0 | 5124 (15.79) |
|  | 26 | Pneumonia | 2473 | Yes = 1 No = 0 Missing if "prefer not to answer" for data-field 2473. | 375 (1.16) |
|  |  |  | 20002 |  |  |
|  | 27 | Chronic bronchitis/emphysema | 6152 | Yes = 1 No = 0 Missing if "prefer not to answer" for data-field 6152. | 234 (0.72) |
|  |  |  | 20002 |  |  |
|  | 28 | Asthma | 6152 | Yes = 1 No = 0 Missing if "prefer not to answer" for data-field 6152. | 3633 (11.20) |
|  |  |  | 20002 |  |  |
| Musculoskeletal | 29 | Rheumatoid arthritis | 2473 | Yes = 1 No = 0 Missing if "prefer not to answer" for data-field 2473. | 167 (0.51) |
|  |  |  | 20002 |  |  |
|  | 30 | Osteoarthritis | 2473 | Yes = 1 No = 0 Missing if "prefer not to answer" for data-field 2473. | 1232 (3.80) |
|  |  |  | 20002 |  |  |
|  | 31 | Gout | 2473 | Yes = 1 No = 0 Missing if "prefer not to answer" for data-field 2473. | 367 (1.13) |
|  |  |  | 20002 |  |  |
|  | 32 | Osteoporosis | 2473 | Yes = 1 No = 0 Missing if "prefer not to answer" for data-field 2473. | 339 (1.04) |
|  |  |  | 20002 |  |  |
| Immunological | 33 | Hayfever, allergic rhinitis or eczema | 6152 | Yes = 1 No = 0 Missing if "prefer not to answer" for data-field 6152. | 8893 (27.40) |
|  |  |  | 20002 |  |  |
|  | 34 | Psoriasis | 2473 | Yes = 1 No = 0 Missing if "prefer not to answer" for data-field 2473. | 308 (0.95) |
|  |  |  | 20002 |  |  |
| Cancer | 35 | Any cancer diagnosis | 2453 | Yes = 1 No = 0 Missing if "prefer not to answer" for data-field 2453. | 2512 (7.74) |
|  |  |  | 134 |  |  |
|  | 36 | Multiple cancers diagnosed (number reported) | 134 | Range from 0 to 6; No cancer or single cancer = 0 Multiple cancers = 1 | 96 (0.30) |
| Pain | 37 | Chest pain | 2335 | Yes = 1 No = 0 | 4285 (13.20) |
|  | 38 | Head and/or neck pain | 6159 | Yes (combining responses to pain in head and neck/shoulders) = 1 No = 0 | 9702 (29.90) |
|  | 39 | Back pain | 6159 | Yes = 1 No = 0 | 6914 (21.39) |
|  | 40 | Stomach/abdominal pain | 6159 | Yes = 1 No = 0 | 2098 (6.47) |
|  | 41 | Hip pain | 6159 | Yes = 1 No = 0 | 2611 (8.05) |
|  | 42 | Knee pain | 6159 | Yes = 1 No = 0 | 5656 (17.43) |
|  | 43 | Whole-body pain | 6159 | Yes = 1 No = 0 | 229 (0.71) |
|  | 44 | Facial pain | 6159 | Yes = 1 No = 0 | 426 (1.31) |
|  | 45 | Sciatica | 2473 | Yes = 1 No = 0 Missing if "prefer not to answer" for data-field 2473. | 189 (0.58) |
|  |  |  | 20002 |  |  |
| Gastrointestinal | 46 | Gastric reflux | 2473 | Yes = 1 No = 0 Missing if "prefer not to answer" for data-field 2473. | 1019 (3.14) |
|  |  |  | 20002 |  |  |
|  | 47 | Hiatus hernia | 2473 | Yes = 1 No = 0 Missing if "prefer not to answer" for data-field 2473. | 494 (1.52) |
|  |  |  | 20002 |  |  |
|  | 48 | Gall stones | 2473 | Yes = 1 No = 0 Missing if "prefer not to answer" for data-field 2473. | 447 (1.38) |
|  |  |  | 20002 |  |  |
|  | 49 | Diverticulitis | 2473 | Yes = 1 No = 0 Missing if "prefer not to answer" for data-field 2473. | 260 (0.80) |
|  |  |  | 20002 |  |  |

**Table S4.** Definition and list of long-term morbidities.

|  | **Morbidity grouping*** | **Conditions included** | **Field ID** |
| --- | --- | --- | --- |
| 1 | Hypertension | Hypertension | 1065 |
|  |  | Essential hypertension | 1072 |
| 2 | Coronary heart disease | Heart attack/MI | 1075 |
|  |  | Angina | 1074 |
| 3 | Diabetes | Diabetic nephropathy | 1607 |
|  |  | Diabetic neuropathy/ulcers | 1468 |
|  |  | Diabetes | 1220 |
|  |  | Type 1 diabetes | 1222 |
|  |  | Type 2 diabetes | 1223 |
|  |  | Diabetic eye disease | 1276 |
| 4 | Stroke/TIA | Stroke | 1081 |
|  |  | TIA | 1082 |
|  |  | Subarachnoid haemorrhage | 1086 |
|  |  | Brain haemorrhage | 1491 |
|  |  | Ischaemic stroke | 1583 |
| 5 | Atrial fibrillation | Atrial fibrillation | 1471 |
| 6 | Heart failure | Cardiomyopathy | 1079 |
|  |  | Hypertrophic cardiomyopathy | 1588 |
|  |  | Heart failure/pulmonary oedema | 1076 |
| 7 | Peripheral vascular disease | Peripheral vascular disease | 1067 |
|  |  | Leg claudication/intermittent claudication | 1087 |
| 8 | COPD | COPD/Chronic obstructive pulmonary disease | 1112 |
|  |  | Emphysema/Chronic bronchitis | 1113 |
|  |  | Emphysema | 1472 |
| 9 | Asthma | Asthma | 1111 |
| 10 | Bronchiectasis | Bronchiectasis | 1114 |
| 11 | Cancer* | “yes”/”no” to “have you ever had cancer?” |  |
| 12 | Dyspepsia | Gastro-oesophageal reflux (GORD) | 1138 |
|  |  | Oesophagitis/Barrett’s oesophagus | 1139 |
|  |  | Gastric stomach ulcers | 1142 |
|  |  | Gastric erosions/gastritis | 1143 |
|  |  | Duodenal ulcer | 1457 |
|  |  | Dyspepsia/indigestion | 1510 |
|  |  | Hiatus hernia | 1474 |
|  |  | Helicobacter pylori | 1442 |
| 13 | Diverticular disease | Diverticular disease/diverticulitis | 1458 |
| 14 | Irritable bowel syndrome | Irritable bowel syndrome | 1154 |
| 15 | Chronic liver disease | Oesophageal varices | 1141 |
|  |  | Non infective hepatitis | 1157 |
|  |  | Liver failure/cirrhosis | 1158 |
|  |  | Primary biliary cirrhosis | 1506 |
| 16 | Inflammatory bowel disease | Inflammatory bowel disease | 1461 |
|  |  | Crohn’s disease | 1462 |
|  |  | Ulcerative colitis | 1463 |
| 17 | Constipation | Constipation | 1599 |
| 18 | Viral hepatitis | Hepatitis B | 1579 |
|  |  | Hepatitis C | 1580 |
|  |  | Hepatitis D | 1581 |
| 19 | Depression | Depression | 1286 |
|  |  | Postnatal depression | 1531 |
| 20 | Anxiety | Anxiety/panic attacks | 1287 |
|  |  | Nervous breakdown | 1288 |
|  |  | Post-traumatic stress disorder | 1469 |
|  |  | Obsessive compulsive disorder | 1615 |
|  |  | Stress | 1614 |
|  |  | Insomnia | 1616 |
|  |  | Psychological/psychiatric problem | 1243 |
| 21 | Schizophrenia/Bipolar affective disorder | Scizophrenia | 1289 |
|  |  | Mania | 1291 |
|  |  | Bipolar disorder | 1291 |
|  |  | Manic depression | 1291 |
| 22 | Connective tissue diseases | Myositis/myopathy | 1322 |
|  |  | Systemic lupus erythematosus/SLE | 1381 |
|  |  | Connective tissue disorder | 1373 |
|  |  | Sjogren’s syndrome.sicca syndrome | 1382 |
|  |  | Dermatopolymyositis | 1383 |
|  |  | Scloeroderma/systemic sclerosis | 1384 |
|  |  | Rheumatoid arthritis | 1464 |
|  |  | Psoriatic arthropathy | 1477 |
|  |  | Dermatomyositis | 1480 |
|  |  | Polymyositis | 1481 |
|  |  | Polymyalgia rheumatica | 1377 |
| 23 | Painful conditions | Back pain | 1534 |
|  |  | Joint pain | 1537 |
|  |  | Headaches (not migraine) | 1436 |
|  |  | Sciatica | 1476 |
|  |  | Plantar fasciitis | 1540 |
|  |  | Carpal tunnel syndrome | 1541 |
|  |  | Fibromyalgia | 1542 |
|  |  | Arthritis | 1538 |
|  |  | Shingles | 1573 |
|  |  | Disc problem | 1532 |
|  |  | Prolapsed disc/slipped disc | 1312 |
|  |  | Spine arthritis/spondylitis | 1311 |
|  |  | Ankylosing spondylitis | 1313 |
|  |  | Back problem | 1294 |
|  |  | Osteoarthritis | 1465 |
|  |  | Gout | 1466 |
|  |  | Cervical spondylosis | 1478 |
|  |  | Trigeminal neuralgia | 1523 |
|  |  | Disc degeneration | 1533 |
|  |  | Trapped nerve/compressed nerve | 1257 |
| 24 | Osteoporosis | Osteoporosis | 1309 |
| 25 | Thyroid disorders | Thyroid problem (not cancer） | 1224 |
|  |  | Hyperthroidism/thyrotoxicosis | 1225 |
|  |  | Hypothyroidism/myxoedema | 1226 |
|  |  | Grave’s disease | 1522 |
|  |  | Thyroid goitre | 1610 |
|  |  | Thyroititis | 1428 |
| 26 | Alcohol problems | Alcohol dependency | 1408 |
|  |  | Alcoholic liver disease/alcoholic cirrhosis | 1604 |
| 27 | Chronic kidney disease | Polycystic kidney | 1427 |
|  |  | Diabetic nephropathy | 1607 |
|  |  | Renal/kidney failure | 1192 |
|  |  | Renal failure requiring dialysis | 1193 |
|  |  | Renal failure not requiring dialysis | 1194 |
|  |  | Kidney nephropathy | 1519 |
|  |  | Immunoglobulin A (IgA) nephropathy | 1520 |
| 28 | Prostate disorders | Prostate problem (not cancer) | 1207 |
|  |  | Enlarged prostate | 1396 |
|  |  | Benign prostatic hypertrophy | 1516 |
| 29 | Glaucoma | Glaucoma | 1277 |
| 30 | Epilepsy | Epilepsy | 1264 |
| 31 | Dementia | Dementia/Alzheimer/cognitive impairment | 1263 |
| 32 | Psoriasis or eczema | Eczema/dermatitis | 1452 |
|  |  | Psoriasis | 1453 |
| 33 | Migraine | Migraine | 1265 |
| 34 | Chronic sinusitis | Chronic sinusitis | 1416 |
| 35 | Anorexia or bulimia | Anorexia, bulimia/other eating disorder | 1470 |
| 36 | Parkinson’s disease | Parkinson’s disease | 1262 |
| 37 | Multiple sclerosis | Multiple sclerosis | 1261 |
| 38 | Chronic fatigue syndrome | Chronic fatigue syndrome | 1482 |
| 39 | Endometriosis | Endometriosis | 1402 |
| 40 | Meniere disease | Meniere disease | 1421 |
| 41 | Pernicious anaemia | Pernicious anaemia | 1331 |
| 42 | Polycystic ovaries | Polycystic ovaries | 1350 |

*Self-report lifetime diagnosis by doctor recorded by interview (UK Biobank data field ID: 20002), except cancer diagnosis which was reported by touch-screen questionnaire. The list of disease groupings was based on Barnett et al (2012).

**Table S5**. Threshold effect analysis of the relationship of arterial stiffness index and probable sarcopenia

| **Arterial Stiffness Index (m/s)** | **Adjusted Model** | |
| --- | --- | --- |
|  | HR (95%CI) | *P* value |
| <13.9 | 1.041 (1.021,1.063) | < 0.001 |
| ≥13.9 | 1.021 (0.886,1.177) | 0.772 |
| Likelihood ratio test |  | 0.655 |

HR, hazard ratio; CI, confidence interval; Adjusted for Model 3.

**Table S6.** Mediating effect of biological age acceleration on the association between arterial stiffness index and probable sarcopenia

| **Item** | **HR** | **Lower** | **Upper** | ***P* value** |
| --- | --- | --- | --- | --- |
| Natural Direct Effect | 1.0321 | 1.016 | 1.0481 | < 0.001 |
| Natural indirect Effect | 1.0029 | 1.002 | 1.0043 | < 0.001 |
| Total Effect | 1.0351 | 1.0192 | 1.051 | < 0.001 |
| Proportion Mediated | 0.0866 | 0.0481 | 0.1734 | < 0.001 |

**Table S7.** Mediating effect of biological age acceleration on the association between arterial stiffness index and sarcopenia

| **Item** | **HR** | **Lower** | **Upper** | ***P* value** |
| --- | --- | --- | --- | --- |
| Natural Direct Effect | 1.0417 | 1.0022 | 1.0783 | 0.04 |
| Natural indirect Effect | 1.0093 | 1.0063 | 1.0128 | < 0.001 |
| Total Effect | 1.0514 | 1.0111 | 1.087 | 0.02 |
| Proportion Mediated | 0.1885 | 0.0932 | 0.6722 | 0.02 |

**Table S8.** The numbers and percentages of incident probable sarcopenia and sarcopenia cases according to frailty status

| **Variables** | **Non-frailty** | **Frailty** |
| --- | --- | --- |
| **Probable sarcopenia, n (%)** |  |  |
| No | 29,169 (94.3) | 1,381 (90.2) |
| Yes | 1,751 (5.7) | 150 (9.8) |
| **Sarcopenia, n (%)** |  |  |
| No | 30,672 (99.2) | 1,502 (98.1) |
| Yes | 248 (0.8) | 29 (1.9) |

**Table S9**. Threshold effect analysis of the relationship of arterial stiffness index and probable sarcopenia in participants without frailty

| **Arterial Stiffness Index (m/s)** | **Adjusted Model** | |
| --- | --- | --- |
|  | HR (95%CI) | *P* value |
| <13.5 | 1.052 (1.03,1.076) | < 0.001 |
| ≥13.5 | 1.057 (0.932,1.198) | 0.3905 |
| Likelihood ratio test |  | 0.508 |

HR, hazard ratio; CI, confidence interval; Adjusted for Model 3.

**Table S10.** Associations of arterial stiffness index with incident possible sarcopenia and sarcopenia in competing risk models

| **Variable (events/total)** | **Model 1** | | **Model 2** | | **Model 3** | |
| --- | --- | --- | --- | --- | --- | --- |
|  | **SHR (95% CI)** | ***P* value** | **SHR (95% CI)** | ***P* value** | **SHR (95% CI)** | ***P* value** |
| **Possible sarcopenia** |  |  |  |  |  |  |
| ASI increase per 1 m/s | 1.03 (1.02, 1.05) | <0.001 | 1.04 (1.02, 1.05) | <0.001 | 1.03 (1.02, 1.05) | <0.001 |
| Tertile 1 (514/10,817) | 1(Ref) |  | 1(Ref) |  | 1(Ref) |  |
| Tertile 2 (614/10,812) | 1.25 (1.11, 1.41) | <0.001 | 1.26 (1.12, 1.42) | <0.001 | 1.25 (1.11, 1.4) | <0.001 |
| Tertile 3 (773/10,822) | 1.36 (1.21, 1.53) | <0.001 | 1.38 (1.23, 1.55) | <0.001 | 1.35 (1.2, 1.52) | <0.001 |
| *P* for trend |  | <0.001 |  | <0.001 |  | <0.001 |
| **Sarcopenia** |  |  |  |  |  |  |
| ASI increase per 1 m/s | 1.05 (1.01, 1.09) | 0.01 | 1.05 (1.01, 1.09) | 0.009 | 1.05 (1.01, 1.09) | 0.015 |
| Tertile 1 (49/10,817) | 1(Ref) |  | 1(Ref) |  | 1(Ref) |  |
| Tertile 2 (90/10,812) | 1.51 (1.07, 2.13) | 0.02 | 1.51 (1.07, 2.14) | 0.02 | 1.44 (1.02, 2.04) | 0.04 |
| Tertile 3 (138/10,822) | 1.59 (1.15, 2.2) | 0.005 | 1.6 (1.15, 2.22) | 0.005 | 1.54 (1.11, 2.14) | 0.01 |
| *P* for trend |  | 0.007 |  | 0.007 | 1.21 (1.04, 1.41) | 0.013 |

Model 1 was adjusted for age and sex; Model 2 was adjusted for ethnic groups, drinking status, and smoking status; Model 3 was further adjusted for educational level, Townsend deprivation index, and number of long-term conditions. ASI, arterial stiffness index; SHR, subdistribution hazard ratio; CI, confidence interval; Ref, reference. Subdistribution hazard ratios were estimated using Fine–Gray models accounting for the competing risk of death.

**Table S11.** Associations of arterial stiffness index with incident possible sarcopenia and sarcopenia in the complete-case analysis

| **Variable (events/total)** | **Model 1** | | **Model 2** | | **Model 3** | |
| --- | --- | --- | --- | --- | --- | --- |
|  | **HR (95% CI)** | ***P* value** | **HR (95% CI)** | ***P* value** | **HR (95% CI)** | ***P* value** |
| **Possible sarcopenia** |  |  |  |  |  |  |
| ASI increase per 1 m/s | 1.04 (1.02, 1.06) | <0.001 | 1.04 (1.03, 1.06) | <0.001 | 1.04 (1.02, 1.05) | <0.001 |
| Tertile 1 | 1(Ref) |  | 1(Ref) |  | 1(Ref) |  |
| Tertile 2 | 1.3 (1.14, 1.47) | <0.001 | 1.31 (1.15, 1.48) | <0.001 | 1.29 (1.14, 1.47) | <0.001 |
| Tertile 3 | 1.44 (1.27, 1.62) | <0.001 | 1.46 (1.29, 1.65) | <0.001 | 1.43 (1.26, 1.61) | <0.001 |
| *P* for trend |  | <0.001 |  | <0.001 |  | <0.001 |
| **Sarcopenia** |  |  |  |  |  |  |
| ASI increase per 1 m/s | 1.06 (1.02, 1.11) | 0.003 | 1.06 (1.02, 1.11) | 0.003 | 1.06 (1.02, 1.1) | 0.004 |
| Tertile 1 | 1(Ref) |  | 1(Ref) |  | 1(Ref) |  |
| Tertile 2 | 1.44 (0.99, 2.11) | 0.058 | 1.45 (0.99, 2.12) | 0.056 | 1.39 (0.95, 2.03) | 0.09 |
| Tertile 3 | 1.71 (1.2, 2.44) | 0.003 | 1.72 (1.21, 2.46) | 0.003 | 1.67 (1.17, 2.38) | 0.005 |
| *P* for trend |  | 0.003 |  | 0.003 |  | 0.005 |

Model 1 was adjusted for age and sex; Model 2 was adjusted for ethnic groups, drinking status, and smoking status; Model 3 was further adjusted for educational level, Townsend deprivation index, and number of long-term conditions. ASI, arterial stiffness index; HR, hazard ratio; CI, confidence interval; Ref, reference. Hazard ratios were estimated using multivariable Cox proportional hazards models.

**Table S12.** Stratified analysis of arterial stiffness index and incident possible sarcopenia

| **Variable** | **HR (95%CI)** | ***P* value** | ***P* for interaction** |
| --- | --- | --- | --- |
| Sex |  |  | 0.868 |
| Female | 1.04 (1.02, 1.06) | <0.001 |  |
| Male | 1.03 (1.01, 1.06) | 0.011 |  |
| Age group |  |  |  |
| <65 years | 1.03 (1.02, 1.05) | <0.001 | 0.833 |
| ≥65 years | 1.03 (1, 1.07) | 0.025 |  |
| Hypertension group |  |  | 0.932 |
| No | 1.04 (1.02, 1.05) | <0.001 |  |
| Yes | 1.03 (1, 1.06) | 0.031 |  |
| Coronary heart disease group |  |  | 0.465 |
| No | 1.04 (1.02, 1.05) | <0.001 |  |
| Yes | 1.01 (0.95, 1.08) | 0.664 |  |
| Dislipidemia |  |  |  |
| No | 1.04 (1.02, 1.06) | <0.001 | 0.484 |
| Yes | 1.02 (0.98, 1.05) | 0.288 |  |
| Heart failure |  |  |  |
| No | 1.03 (1.02, 1.05) | <0.001 | 0.279 |
| Yes | 1.12 (0.78, 1.59) | 0.548 |  |
| Depression |  |  |  |
| No | 1.04 (1.02, 1.05) | <0.001 | 0.191 |
| Yes | 0.99 (0.92, 1.06) | 0.762 |  |
| Connective tissue disease |  |  |  |
| No | 1.04 (1.02, 1.05) | <0.001 | 0.717 |
| Yes | 1.02 (0.93, 1.12) | 0.663 |  |
| Osteoporosis |  |  |  |
| No | 1.03 (1.02, 1.05) | <0.001 | 0.842 |
| Yes | 1.03 (0.92, 1.14) | 0.644 |  |
| Dementia |  |  |  |
| No | 1.03 (1.02, 1.05) | <0.001 | 0.126 |
| Yes | 0.12 (0, Inf) | 0.999 |  |
| COPD |  |  |  |
| No | 1.04 (1.02, 1.05) | <0.001 | 0.622 |
| Yes | 1.12 (0.98, 1.28) | 0.106 |  |
| Fractures |  |  |  |
| No | 1.03 (1.02, 1.05) | <0.001 | 0.582 |
| Yes | 1.04 (1, 1.09) | 0.072 |  |
| Falls in last year |  |  |  |
| No | 1.04 (1.02, 1.06) | <0.001 | 0.498 |
| Yes | 1.02 (0.98, 1.06) | 0.256 |  |

Adjusted for Model 3. Ref, reference.

**Table S13.** Stratified analysis of arterial stiffness index and incident sarcopenia

| **Variable** | **HR (95%CI)** | ***P* value** | ***P* for interaction** |
| --- | --- | --- | --- |
| Sex |  |  | 0.428 |
| Female | 1.01 (0.94, 1.09) | 0.7 |  |
| Male | 1.06 (1.01, 1.11) | 0.012 |  |
| Age group |  |  | 0.055 |
| <65 years | 1.02 (0.97, 1.07) | 0.464 |  |
| ≥65 years | 1.1 (1.03, 1.17) | 0.004 |  |
| Hypertension group |  |  | 0.053 |
| Non hypertension | 1.07 (1.01, 1.12) | 0.013 |  |
| Hypertension | 1.02 (0.96, 1.08) | 0.532 |  |
| Coronary heart disease group |  |  | 0.056 |
| Non coronary heart disease | 1.06 (1.02, 1.1) | 0.004 |  |
| Coronary heart disease | 0.93 (0.81, 1.07) | 0.303 |  |
| Dislipidemia |  |  |  |
| No | 1.07 (1.03, 1.12) | 0.002 | 0.051 |
| Yes | 0.99 (0.92, 1.07) | 0.843 |  |
| Heart failure |  |  |  |
| No | 1.05 (1.01, 1.09) | 0.02 | 0.187 |
| Yes | 2 (0, Inf) | 0.999 |  |
| Depression |  |  |  |
| No | 1.06 (1.01, 1.1) | 0.008 | 0.199 |
| Yes | 0.94 (0.78, 1.13) | 0.491 |  |
| Connective tissue disease |  |  |  |
| No | 1.05 (1.01, 1.09) | 0.013 | 0.813 |
| Yes | 1.07 (0.88, 1.3) | 0.478 |  |
| Osteoporosis |  |  |  |
| No | 1.05 (1.01, 1.09) | 0.015 | 0.914 |
| Yes | 1.05 (0.76, 1.44) | 0.771 |  |
| Dementia |  |  |  |
| No | 1.05 (1.01, 1.09) | 0.015 | 1 |
| Yes | 10.43 (0, Inf) | 0.999 |  |
| COPD |  |  |  |
| No | 1.05 (1.01, 1.09) | 0.015 | 0.86 |
| Yes | 1.14 (0.86, 1.51) | 0.365 |  |
| Fractures |  |  |  |
| No | 1.05 (1.01, 1.09) | 0.018 | 0.892 |
| Yes | 1.03 (0.88, 1.2) | 0.742 |  |
| Falls in last year |  |  |  |
| No | 1.07 (1.03, 1.12) | 0.001 | 0.091 |
| Yes | 1 (0.89, 1.11) | 0.936 |  |

Adjusted for Model 3. Ref, reference.

**REFERENCES**

1. Cruz-Jentoft AJ, Bahat G, Bauer J, Boirie Y, Bruyere O, Cederholm T, et al. Sarcopenia: revised European consensus on definition and diagnosis. Age Ageing. 2019;48(1):16-31. doi: 10.1093/ageing/afy169.

2. Simsek SM, Gok G, Corut R, Kilic N. Arterial Stiffness in Pre-COPD/PRISm, and COPD Stages: A Cross-Sectional Assessment of Hemodynamic Determinants. Int J Chron Obstruct Pulmon Dis. 2026;21:594006. doi: 10.2147/COPD.S594006.

3. Alonso-Diaz J, Gomez-Sanchez M, Arjol D, Gonzalez-Sanchez S, Rodriguez-Sanchez E, Garcia-Ortiz L, et al. Dietary Fiber Is Inversely Associated with Central Arterial Stiffness Progression, While Alcohol and Iron Intake Are Positively Associated with CAVI: A 5-Year Longitudinal Study. Nutrients. 2026;18(9). doi: 10.3390/nu18091314.

4. Talevski J, Brennan-Olsen S, Bird S, Vogrin S, Beauchamp A, Fatima M, et al. Bone, muscle, and physical function measures in older adults according to levels of social disadvantage: a cross-sectional study. Arch Osteoporos. 2026;21(1):29. doi: 10.1007/s11657-026-01661-5.

5. Sun B, Ruan Z, Xiao W, Wang J, Liu Y, Wang S, et al. Association of multimorbidity and its patterns with incident possible sarcopenia among middle-aged and older adults: findings from a national prospective cohort study in China. BMC Musculoskelet Disord. 2026;27(1). doi: 10.1186/s12891-026-09750-9.
